# Diverging Discourses: Animal Health Challenges and Veterinary Care in Northern Uganda

**DOI:** 10.3389/fvets.2022.773903

**Published:** 2022-03-10

**Authors:** Anna Arvidsson, Klara Fischer, Kjell Hansen, Susanna Sternberg-Lewerin, Erika Chenais

**Affiliations:** ^1^Department of Urban and Rural Development, Swedish University of Agricultural Sciences, Uppsala, Sweden; ^2^Department of Biomedical Science and Veterinary Public Health, Swedish University of Agricultural Sciences, Uppsala, Sweden; ^3^Department of Disease Control and Epidemiology, National Veterinary Institute (SVA), Uppsala, Sweden

**Keywords:** discourse coalitions, Africa, disease prevention, animal health services, paraprofessionals

## Abstract

People in northern Uganda are currently rebuilding their lives after a lengthy period of conflict. To facilitate this, the Ugandan government and donors have promoted investment in pigs as an important strategy for generating income quickly and ensuring livelihood security. In this context, animal health issues are an acknowledged challenge, creating uncertainty for animal owners who risk losing both their animals and income. This paper draws on policy documents guiding the veterinary sector, interviews with faculty staff at Makerere University and with veterinarians and paraprofessionals in northern Uganda, and ethnographic fieldwork in smallholder communities. The aims of this study were to contribute to an understanding of the structure of veterinary support and its dominant development narratives in policy and veterinary education and of the way in which dominant discourses and practices affect smallholders' ability to treat sick animals. Particular attention was paid to the role of paraprofessionals, here referring to actors with varied levels of training who provide animal health services mainly in rural areas. The results suggest that veterinary researchers, field veterinarians and government officials in agricultural policy share a common discourse in which making smallholders more business-minded and commercializing smallholder production are important elements in reducing rural poverty in Uganda. This way of framing smallholder livestock production overlooks other important challenges faced by smallholders in their livestock production, as well as alternative views of agricultural development. The public veterinary sector is massively under-resourced; thus while inadequately trained paraprofessionals and insufficient veterinary support currently present a risks to animal health, paraprofessionals fulfill an important role for smallholders unable to access the public veterinary sector. The dominant discourse framing paraprofessionals as “quacks” tends to downplay how important they are to smallholders by mainly highlighting the negative outcomes for animal healthcare resulting from their lack of formalized training. The conclusions of this study are that both animal health and smallholders' livelihoods would benefit from closer collaboration between veterinarians and paraprofessionals and from a better understanding of smallholders' needs.

## Introduction and Background

This study explored aspects of animal health challenges in a setting where animal production is a key feature in many people's lives. Agriculture remains the cornerstone of Uganda's economy and contributes ~28% of total GDP. Over 70% of the population are engaged in agricultural activities, mainly for subsistence (1). The Ugandan government sees the shift from subsistence farming to commercial agriculture as a key strategy for reducing widespread poverty in the country (2). In the general efforts to reduce poverty through the commercialization of agriculture, the Ugandan government and donors have particularly focused on livestock as an opportunity for smallholders. Pigs especially have attracted interest due to their short generation interval, minimal space requirement and rapid multiplication rates (3, 4)^1^. However, their potential as a route out of poverty is limited by, among other things, disease and health problems (5–8). Smallholders have limited access to veterinary services, with the main providers often being paraprofessionals (9, 10). The quantity and quality of paraprofessional training varies, with the result that they can offer important advice and support to smallholders as well as cause severe animal suffering due to inappropriate treatment (9, 11). Consequently, even seemingly minor and non-fatal problems, such as worms and diarrhea, significantly constrain production and lead to livestock loss (12). In light of this, veterinary services play a key role in the government's efforts to reduce poverty through agricultural commercialization in general and pig production in particular.

In this paper we examine the government's focus on agriculture commercialization, concentrating on livestock production as a route out of poverty, and the factors perceived to influence smallholders' achievement of this goal. The aims were to acquire a better understanding of how veterinary actors and central policy documents frame the issues that hinder smallholder livestock production and the solutions offered (13, 14). Particular attention was paid to the role of paraprofessionals and how they fulfill an important function in giving advice to smallholders, but are nevertheless constructed as a problem in the dominant discourse. Applying the analytical concepts of storylines, narratives and discourse coalitions (15, 16), the intention was to establish how smallholder agricultural development is framed and the extent to which the challenges perceived by smallholders are addressed in policy and veterinary education.

### The Rise of Pig Production in Northern Uganda

While pork used to be taboo among large sections of the Ugandan population (17–19), demand is now growing and pig production nationwide has increased (20). Most pigs are kept in traditional smallholder systems, in which the animals are free-roaming, tethered or confined to a pig sty (21). In 1959, the country had 15,669 pigs [(22), p. 95]; by 2018 that number had exceeded 4 million (23). Today, Uganda has the highest per capita consumption of pork in East Africa, with average consumption of 3.4 kg per person per year (24). Therefore, pigs are now recognized as an important source of income for smallholder farmers and described as a potential route out of poverty (25–27). Policymakers and researchers identify the main obstacle to the upscaling of pig production to be African swine fever (ASF) (28), a haemorrhagic infectious disease with a very high mortality rate in domestic pigs (29) and that is endemic in Uganda (30). There is no cure or vaccine for it, making disease prevention the only strategy to limit its spread (31).

Geographically, this study focused on northern Uganda. The majority of people in this area belong to the Acholi people and speak Luo. The region is particularly badly affected by poverty and marginalization due to repeated conflicts resulting in a loss of livelihood assets in the past, including livestock (32). The most recent conflict in this part of the country started in 1986 and was mainly fought by the Ugandan government and the Lord's Resistance Army (LRA) rebel group. Approximately 1.8 million people were displaced during the conflict, and the vast majority of the population was forced to stay in camps for internally displaced people (so-called IDP camps) run by the government (33). Opportunities for agricultural activities in the camps were very limited and many smallholders lost their animals either to fighting forces or cattle raiders who exploited the opportunities resulting from instability during the conflict (34). People started returning to their former villages between 2006 and 2009, and slowly resumed animal keeping and cultivation (35). The loss of livestock during the conflict left many Acholi considerably worse off than they were prior to the conflict. Against this backdrop, the government and donors promoted pig production as a quick method of poverty mitigation for farmers who had few other resources (21).

The smallholders in this study live in two different villages (referred here as to village A and village B) located in Nwoya district, northern Uganda. Village A can only be reached on dirt roads, ~30–40 min by motorbike from the main road. The small village center has some local businesses, including hairdressers, local bars and a slaughter place. Village B served as an IDP camp during the conflict and is located alongside the main (tarred) road connecting the nearest city of Gulu with the capital Kampala. Village B therefore has better connections with urban markets. Most smallholders in this study had access to small plots of land for crop cultivation. Those engaged in pig production generally kept one to five pigs (local, cross and exotic breeds) that were mainly fed with cassava, swill, maize or rice bran. Access to pharmaceuticals and formal livestock markets generally required travel to Gulu city or a larger town that few of the studied smallholders could afford.

### Structural Adjustment and the Downsizing of the Veterinary Sector in Uganda

To better understand the current situation as regards uncertain access to veterinary services and the role of paraprofessionals, it is useful to explore some of the recent history of agricultural policy. In the late 1980's and early 1990's, the Ugandan government adopted structural adjustment programmes (SAPs) under conditions attached to loans from the World Bank and the International Monetary Fund. This led to major structural changes in the provision of veterinary services. Loan conditionality commonly included the downsizing of public services, liberalization of markets, privatization of public enterprises and major reductions in government subsidies to agriculture, export promotion and other policies aimed at boosting economic growth (36–38). In Uganda, the previous government-led public veterinary sector was transformed during this period into a decentralized and privatized structure of clinical veterinary services, which included the downscaling of public services (11, 39, 40). These changes, among other things, led the Ugandan government to adopt a reactive service delivery rather than a proactive one (11, 27). For example, instead of being part of a preventive practice, vaccinations are generally administered by the public veterinary sector during disease outbreaks (27). The Ministry of Agriculture, Animal Industry and Fisheries (MAAIF) remains responsible for vaccinating animals against epidemic diseases, imposing quarantines and controlling tsetse flies, and deliver those services for free, but clinical services, breeding and spraying for tick control are now privatized (41). Field veterinarians and paraprofessionals working in the public sector also provide clinical services for which farmers are expected to pay (11). In parallel with the downscaling of the public veterinary sector, state subsidies in animal healthcare were withdrawn and the veterinary drug market liberalized (42). The simultaneous processes of reduced access to veterinarians and increased access to pharmaceuticals through private retailers resulted in both smallholders and paraprofessionals frequently turning to drug retailers for animal health information. However, the differing levels of competence among these retailers in combination with the circulation of counterfeit, diluted and expired pharmaceuticals often exacerbated rather than addressed the issue of lack of access to trained veterinarians (28). Another consequence of this privatization is that many paraprofessional actors providing veterinary services to farmers today do so without sufficient supervision, making it hard to ensure that accurate advice is given and regulations are followed (43, 44).

### The Role of Paraprofessionals in Uganda's Veterinary Sector

In today's free market of veterinary service providers, veterinarians operating under MAAIF and local governments at district level work with private practitioners, including veterinarians and paraprofessionals (7). In this specific situation, paraprofessionals are authorized by the Veterinary Statutory Body to perform certain tasks under the supervision and responsibility of a veterinarian (45). The approach of letting paraprofessionals fill a gap previously covered by government veterinary services was initially a response to the failed privatization of veterinary services in many countries in the Global South, and was intended to deal with less complicated animal health problems in communities that had limited access to qualified veterinary care (46). However, paraprofessionals in Uganda rarely work under such supervision and few are mandated by the Veterinary Statutory Body to carry out their work (9). Since the 1990's, there has been no formal institution providing paraprofessionals with specific training in veterinary medicine in Uganda (41). Due to their current unregulated training, paraprofessionals' knowledge varies greatly: some hold a certificate or a diploma in general agriculture or animal management, while others have only had a few months' training through an NGO or no relevant training at all (40, 47).

## Conceptual Framework

Our analysis in this study was based on the assumption that “the political conflict is hidden in the question of what definition is given to the problem, which aspects of social reality are included, and which are left undiscussed” [(16), p. 43]. Based on this guiding idea, the analysis explored how actors within the Ugandan veterinary sector frame the factors hindering smallholder livestock production and what solutions are developed to address these specific problems (13, 14, 48). The discourse analysis aimed to identify narratives on development through livestock production among actors within the Ugandan veterinary sector. It draws on the “social-interactive” discourse theory in which it is claimed that “actors can only make sense of the world by drawing on the terms of the discourses available to them” [(16), p. 53]. It was therefore of interest to explore how veterinary actors talk about and act on animal disease and development in different ways, and how these descriptions and practices reinforce or challenge particular discourses. The term “discourse” here refers to “a specific ensemble of ideas, concepts and categorizations that are produced, reproduced, and transformed in a particular set of practices, and through which meaning is given to physical and social realities” [(16), p. 44]. The term “practices” in the previous sentence is understood here to mean both language and actions. Veterinary work is thus conceptualized in the analysis as an important extension and practical implication of the discourse. Our exploration of how the practices and descriptions of the studied actors support or challenge particular discourses drew on the concept of an “argumentative game.” Actors who engage in an “argumentative game” seek to achieve discursive hegemony by using particular narratives (or storylines, see below) to communicate and seek support for their view of reality [(16), p. 59]. In this argumentative game, actors depend on credibility, acceptability and trust to gain and maintain support for their way of narrating reality and influencing practices (16). Thus, analyzing how actors were given credibility, acceptability and trust by others allowed an understanding of the position given to these actors in the argumentative game. Here, it was also useful to look for truth claims, as these are key for upholding a specific discourse (49, 50). The way that “truths” are constructed in a discourse means that particular worldviews are portrayed as natural and obvious, whereas alternative ways of thinking and acting becomes unthinkable and thereby discredited in the discourse (51).

The way actors actively seek to achieve discursive hegemony by using particular narratives can be analyzed through the concept of storylines. A storyline can be defined as “a generative sort of narrative that allows actors to draw upon various discursive categories to give meaning to specific physical or social phenomena” [(16), p. 56]. In short, a storyline can be understood as “a condensed statement summarizing complex narratives” [(52), p. 61], thus functioning to simplify complex narratives and suggest unity, despite the presence of competing or contradictory narratives. Actors can position themselves using storylines, and thus finding convincing storylines becomes an important form of agency. However, people might not intentionally use storylines as a way of positioning themselves, but rather assume that their way of describing reality is just how things should be thought and talked about.

To succeed in having a perspective dominate in policy not only requires efficient communication strategies, but just as importantly the building of political and economic alliances, for example. Here the concept of “discourse coalitions” is helpful in analyzing how certain actors come together and support the same discourse. A discourse coalition can be defined as “the ensemble of particular storylines, the actors who employ them, and the practices through which the discourse involved exerts its power” [(15), p. 61]. As such, the term “discourse coalition” moves the power analysis beyond text and acknowledges that it also matters who it is who joins particular alliances and supports a certain discourse.

## Materials and Methods

The study draws on policy documents, semi-structured interviews with staff working in veterinary education at Makerere University in Kampala and with field veterinarians and paraprofessionals in Nwoya district, and ethnographic fieldwork in two villages in Nwoya district. Table 1 provides an overview of the qualitative data methods used and Table 2 presents the different animal health service providers mentioned in this paper.

**Table 1:** Overview of qualitative data collection: methods and informants.

**Type of method**	**Category of informants**	**Total no. of participants**
Semi-structured	Staff at Makerere University^a^	5
interview	Field veterinarians and DVO	6
	Paraprofessionals^b^	6
	Smallholders	70^c^
Focus group discussion & ranking exercise^d^	Smallholders	43
Questionnaire in survey	Smallholders	101
Paticipant observation^e^	(N/A) village life and farming	(N/A)
	Field veterinarians	1
	Paraprofessionals	1

a *All interviews were conducted online via video link*.

b *Five out of six interviews were conducted over the telephone with a field assistant on site translating*.

c *The total number of 70 refers to households and not to individuals, with all but two households located in village A*.

d *All but one focus group discussion included a ranking exercise*.

e *Participant observation in study villages A and B was not focused on following particular smallholders, therefore no specific number is given here. See Section Participant Observation for further details*.

**Table 2:** Description of formal and informal animal health service provider terms mentioned in this paper.

**Term**	**Type of employment**	**Definition**
Veterinary field officers/field veterinarians	Public and private sector	Individuals with a degree in veterinary medicine from a veterinary institution
District veterinary officer (DVO)	Public sector	Individuals responsible for government-led veterinary work at sub-county level
Faculty staff members	Public sector	Individuals working in veterinary education at Makerere University in Kampala, Uganda
Paraprofessionals	Public and/or private sector	Used here as an umbrella term to describe individuals who: i) have received formal training at certificate or diploma level in animal health or general agriculture (in the literature often referred to as *paraveterinarians*) ii) have received very limited or no formal training in animal health, but may have acquired knowledge through practical experience (in the literature often referred to as *community animal health workers)*
Doctors	Public and private sector	Individuals with a degree in veterinary medicine from a veterinary institution (veterinarians), referred to by smallholders and paraprofessionals in this study as “doctors”
Scientists	Private sector	Individuals without formal training or a degree in veterinary medicine or animal health, but who other community members may know to be qualified in animal healthcare through their practical experience
Extension workers/staff	Public and/or private sector	An umbrella term used to describe a wide range of actors who assist farmers with crop and/or livestock production
Quacks	Private sector	A term used to describe individuals with limited or no training in animal health/veterinary medicine who provide incorrect advice or treatment under the pretense of being skilled in veterinary medicine

The majority of ethnographic data were collected in village A, where the first author stayed for 4 months with a Ugandan family during fieldwork. Additional data were collected in village B. The distance between the two villages is ~30 km. The first author made detailed notes during all the interviews and focus group discussions. Interviews conducted in English were recorded for the purpose of filling in gaps in the notes, but were not transcribed in detail. Interviews conducted in smallholder communities were simultaneously translated between Luo and English by the field assistants. These interviews were discussed with the field assistants directly after the interviews to fill in gaps and provide clarification. Prior to participating, the interviewees were informed of the purpose of the interview and the expected outcome of the study, asked for their oral consent, and informed that they could withdraw their participation at any time for any reason. The names of the two study villages are not included and the names of all interviewees have been changed to maintain their anonymity. The quotations serve to give life to the findings and should not be taken ad verbatim.

### Policy Documents

The following policy documents that inform the Ugandan livestock and veterinary sector were analyzed to explore discourses on the role of veterinary actors, livestock production and development: the National Agricultural Extension Strategy (NAES) 2016/17-2020/21, the National Agricultural Extension Policy (NAEP) and the Extension Guidelines and Standards, which were all approved by MAAIF in 2016. The Ethical Code of Conduct for Agricultural Extension and Advisory Service Providers approved by MAAIF in 2019, the National Agriculture Policy (NAP) and the National Adaption Plan for the Agricultural Sector (NAP-Ag) approved by MAAIF in 2013 and 2018, respectively, were also included. Several of the above policy documents refer to the Uganda Vision 2040, described as the Ugandan “30-year development master plan” [(53), p. 61], produced by the National Planning Authority and launched by president Yoweri Kaguta Museveni in 2013. This document was therefore also included in the analysis. Finally, to explore correlational and potentially contradictory narratives in national and international policy documents on the livestock sector, the EAC Livestock Policy adopted by the East African Community in 2016 and the report of Business and Livelihoods in African Livestock published by the World Bank in 2014 were also analyzed. The selection of documents was guided by the aim to cover a wide range of veterinary and agricultural policy frameworks and was restricted by the online availability of national policy documents.

### Interviews With Staff at Makerere University

Semi-structured interviews were performed with staff in the veterinary faculty at Makerere University in Kampala with the aim of understanding the structure and content of veterinary education and capture perspectives on veterinary education and extension work. These interviews were conducted remotely via video link in December 2020 and January 2021. The staff work in the School of Veterinary Medicine and Animal Resources (MakSVAR), one of two schools in the College of Veterinary Medicine, Animal Resources and Biosecurity (CoVAB), referred to in this paper as “the veterinary faculty.” The interviewees were selected by a key informant working in the faculty based on the criteria of having broad knowledge of the work of veterinarians and of education at the veterinary faculty, which is the only one in Uganda. In addition, with the aim of capturing broad perspectives on veterinary education and related topics, the staff informants selected have differing educational backgrounds, ranging from a bachelor's degree to a PhD in veterinary medicine. Staff members held the following positions at the veterinary faculty: teaching assistant, senior lecturer, associate professor (two informants) and dean. The interviews were semi-structured, following a pre-defined topic guide (see Annex 1). Interviews were conducted in English (all informants were fluent in English) and lasted between 45 and 90 min.

### Interviews With Field Veterinarians and Paraprofessionals

Veterinarians and paraprofessionals working in Nwoya district were interviewed with the purpose of acquiring a better understanding of veterinary and animal health work in northern Uganda. Field veterinary informants were selected by the district veterinary officer (responsible for government-led veterinary work at sub-county level). To capture perspectives on professional veterinary work that were as broad as possible, all the field veterinarians suggested for inclusion in the study by the district veterinary officer were interviewed. The first paraprofessional informant lived in village A, and additional informants in this category were identified through snowball selection (54), with one paraprofessional suggesting another. With the aim of approaching a variety of actors referred to as paraprofessionals, the paraprofessionals were deliberately asked to recommend both those who were considered competent and those who had a poor reputation and were referred to as “quacks.” The interviews with the veterinary field officers and district veterinary officer were conducted on site in English by the first author (all the informants were fluent in English). One interview with a paraprofessional was conducted on site with a translation between Luo and English. The remaining interviews with paraprofessionals were conducted over the telephone in April and May 2021, with the field assistant on site translating.

### Ethnographic Fieldwork

Smallholder perspectives were captured through ethnographic fieldwork carried out between September and December 2019. The fieldwork was conducted with the purpose of gaining a broad understanding of the role of pigs and the general conditions for livestock production in this setting. The area for ethnographic fieldwork was strategically selected due to reports of ASF in the past and the authors having established research contacts prior to this study. One field assistant lived in village A and the other in village B, but during the ethnographic fieldwork both of them spent most of their time in the village A.

#### Participant Observation

The key method for data collection on smallholders' use of pigs and their perspectives on animal health and access to veterinary services was participant observation (55). The first author stayed with a Ugandan family in village A for 4 months, took part in daily chores and engaged in village life, while continuously taking notes and actively reflecting and asking questions about what she participated in and observed. Participant observation was complemented and triangulated (56) with individual interviews, focus group discussions and a survey, as described below.

#### Individual Interviews With Smallholders

Interviews with smallholder households in study villages A and B were conducted with the purpose of gaining more detailed insights into informants' perspectives of pig production and access to veterinary services. The interviews varied in length, depending on the time the participants had available. In several households, more than one person participated in the interview, and several households were interviewed more than once. Participants were purposively selected on the basis of being household members aged over 18 with previous knowledge of livestock production. The interviews were semi-structured and aimed to capture broad perspectives about smallholders' livestock production. All the interviews were conducted with the help of a field assistant who translated between Luo and English.

#### Focus Group Discussions

With the purpose of capturing a broad range of views on livestock management and animal health issues, six focus group discussions were held with smallholders from village A. Special attention was paid in the focus groups to allowing the participants to steer the discussion toward subjects of interest to them. One field assistant facilitated discussions and another translated between Luo and English. The first author took detailed notes and intervened when clarification was needed. Participants were purposively selected on the basis of being residents of the study village with previous experience of livestock production, as well as having the time and a willingness to participate. Four groups contained both men and women, and two further groups had only women in them. The purpose of the separate women's groups was to ensure that the women could speak freely in discussions that otherwise risked being dominated by the views of male participants. Participants were asked for detailed descriptions of problems with their livestock production, as well as potential ways to prevent or resolve the issues raised. Problems and solutions were written down on a large piece of paper in both English and Luo by the facilitator in front of the group. In five of the focus groups, participants ranked the problems in relation to one other, according to their perceived magnitude (Annex 2).

#### Survey

Based on the initial findings from individual semi-structured interviews and focus group discussions with smallholders, a survey was designed (Annex 3) to quantify smallholders' framing and prioritization of livestock problems, as well as their perception of access to and costs of veterinary services. The survey was written in English and translated into Luo by the field assistant, and was conducted during interviews in which the field assistant, trained by the first author, interviewed respondents in Luo and noted down their answers in English. Respondents were selected based on a mix of purposive and convenience sampling strategies. The inclusion criteria were adult household members who lived a manageable travel distance from the field assistant's home, had previous knowledge of livestock production and were at home during the time of the field assistant's visit. For convenience, the survey (101 responses) was mainly conducted in village B (85 responses) where the field assistant lived, with some additional data collection in village A (16 responses).

### Data Handling and Analysis

Field notes from interviews and focus group discussions were transcribed as soon as possible after each interview. Except for the survey, all material was analyzed by the first author using Nvivo software (QSR International). In the initial stage of the analysis, the first author read all the material thoroughly and inductively categorized the data according to broadly defined codes such as “livestock challenges,” “extension work,” and “quacks.” The content of the broad themes was then reviewed in dialogue with the second and third authors, discussing what was interesting and how this could be interpreted. The conceptual framework (Section Conceptual Framework) was designed and subsequently used in a second round of iterative inductive-deductive process coding for narrower themes and patterns guided by the conceptual framework, but open to emerging themes from the data. Narrower themes included, for example, “knowledge transfer,” “mindset change,” “ASF,” and “entrepreneurship.” This round of analysis also examined the argumentative structure in the textual material and looked for coherent statements and storylines, as well as who expressed them and how they were positioned in relation to other actors and statements. Data from the survey were collected on paper questionnaires by the field assistant and entered into a Microsoft Excel spread sheet by the first author, supported by the fourth and fifth authors, to gain an overview of the data as well as estimate the minimum, maximum and average of the numerical results (Annex 4).

## Results

The findings showed that all the actors in this study agreed that underfunding of the veterinary sector is a real and significant challenge to improving livestock health in the country, as described in the following section. The actors did not agree on the underlying causes or possible solutions to this, however, as described in subsequent sections of the results. These findings illustrate that there is a dominant discourse centered on commercialization of smallholder agriculture as a route out of poverty, which is maintained by a discourse coalition of staff at the veterinary faculty, government officials and, to some extent, field veterinarians. The key features of this dominant discourse are described in Sections Entrepreneurship, Market Orientation and the Coalition Around Them to The Problem With “Quacks”. Paraprofessionals and smallholders challenged this dominant discourse, but the analysis indicated that expressions challenging the dominant discourse were less coherent and did not form a strong united discourse coalition.

### Widespread Agreement on Underfunding of the Veterinary Sector

The analysis of policy documents and interviews with veterinary actors and smallholders confirmed the results of previous studies and showed that all the actors equally acknowledged smallholders' lack of access to veterinary advice as a key issue, particularly in northern Uganda. In policy documents, understaffing and underfunding of the veterinary sector were repeatedly described as real and major problems (57, 58). For example, in 2016, the ratio of extension staff (which includes all government actors who assist farmers with crop and livestock production) to farmers in Uganda at large was more than 1:5,000 compared with the recommended ratio of 1:500 [(57), p. 14)]. Interviews with staff at the veterinary faculty revealed that there were fewer students on the veterinary programme from the northern and eastern regions than from the central and western regions of Uganda. Several members of the veterinary faculty perceived it to be less likely that people from rural, remote areas would be able to afford veterinary education. This was thought to be connected to recent increased competition for government sponsorships, which has made it even harder for poorer families to send a household member to university. According to respondents in the veterinary faculty, it used to be more common to have students with a background of rural poverty; today, however, most students come from better-off families where at least one of the parents has had a higher education.

Related to the above issue, veterinary faculty respondents also emphasized that veterinary students from northern Uganda preferred to take jobs in central Uganda after graduating, since salaries are generally higher closer to the capital and aspirations to a modern lifestyle involve staying in a large city. In addition, according to the veterinary faculty staff and field veterinarians interviewed, the lack of laboratories and work facilities in the public sector made it less attractive to apply for jobs in the northern region. However, both veterinary faculty respondents and field veterinarians believed that this tendency could be changed by recent improvements in salaries in the public veterinary sector.

Field veterinarians working in the public sector were paid a basic salary, but were expected to receive compensation for fuel, material and pharmaceuticals from farmers. Some field veterinarians described how this led them to approach large-scale farmers rather than poor smallholders to ensure that they would be compensated for their work. Several smallholders said that they called the veterinarian as the last resort when nothing else had worked. Field veterinarians explained how this made it difficult for them to be successful, as animals often were very ill and beyond saving by the time they were called. If animals did not recover after being treated, smallholders were sometimes unwilling to pay for their services, something that field veterinarians described as causing them stress and increasingly leading them to focus on large-scale farmers who are more able to pay. When discussing the difficulty smallholders had in accessing veterinary services, field veterinarians generally stressed that veterinary services are demand-driven and that it is the responsibility of smallholders to approach field veterinarians. In the words of field veterinarian Charles: “*You see, if someone is sick, then the person must go to the hospital to see the doctor; the doctor can't know that someone is sick if they stay at home. That's how it works. So, farmers should reach out to us.”* However, several field veterinarians also understood that the problem was connected to previous structural changes in the veterinary sector. In the past, some veterinary services were provided for free, whereas today the public sector has been downscaled and farmers are expected to pay for veterinary services themselves.

### Entrepreneurship, Market Orientation and the Coalition Around Them

When exploring narratives relating to the desired development of agriculture and livestock production, one clear storyline was repeatedly found across all policy documents in slightly different variations: “To transform the sector from subsistence farming to commercial agriculture” [(59), p. 14, (60), p. 34]. The idea that smallholders need to leave the subsistence level of farming in order to become developed was also clearly expressed in policies specifically targeting the veterinary sector with wording such as: “…to provide agricultural extension services in order to support sustained progression of smallholder farmers from subsistence agriculture to market oriented and commercial farming” [(57), p. 3]. This storyline binds together broader narratives of agricultural development with ones about the role of the veterinary profession specifically, and describes an “agricultural revolution” in Uganda (57, 59) where smallholders need to be part of a modernization process, start contributing to economic growth by scaling up their enterprises, and become integrated in the formal liberalized market. This discourse implies that policymakers do not acknowledge any particular strengths of small-scale farming. Instead, what captures the political imagination is promises that large-scale farms will generate capital and play a key role in transforming the livestock sector [(61), p. 46]. This discourse, found in fairly similar versions in the various policy documents, was intertextually connected with “Uganda Vision 2040,” an overarching development policy for the country that aims to provide development paths and strategies whose stated overarching goal is to achieve “a transformed Ugandan society from a peasant to a modern and prosperous country within 30 years” [(2), p. 3]. It further states that “Uganda aspires to transform the agriculture sector from subsistence farming to commercial agriculture” [(2), p. 45], and to achieve this aspiration, the “right attitudes and mindsets” of the population are needed [(2), p. 4].

Investigating the role given to the veterinary sector in the proposed agricultural transformation in more detail, it became clear that there are a number of key issues and associated solutions that guide action in this sector. Apart from an acknowledgment that the sector is understaffed and underfunded, the main reason for smallholders not implementing veterinary policies was framed as a problem of information. The documents suggest that information is a fixed entity that should be packaged and conveyed to smallholders, with the aim of getting smallholders to adopt and adjust their practices in line with the information given, as exemplified in the following quotation stating that the strategic mission of the National Extension Strategy is to “promote application of appropriate information, knowledge and technological innovations for commercialization of agriculture” [(57), p. 16]. That this envisioned change of smallholders into entrepreneurs did not happen in practice was primarily explained as a problem of communication. There were two sides to it: a problem with how the information is communicated by extension workers, leading to the conclusion that “extension workers need to be adequately equipped with content and methodology to deliver them to beneficiaries” [(57), p. 12], and a problem that smallholders do not follow the recommendations, frequently described as an issue with smallholders' mindsets, as also seen in the previous quotation from Uganda Vision 2040. There was no questioning of whether the solutions proposed in the policy fitted with smallholders' wider contexts and practices. Rather, smallholders needed to “change their mindsets” so that they would better appreciate the services provided and better understand the importance of being business-oriented [(62), p. 25]. In addition, claims that smallholders had previously failed to adopt new innovations and technologies (63) led to the conclusion that smallholders needed to be educated and “sensitised.” The essence was that if smallholders could be sensitised, educated and willing to change their attitudes and mindsets, they would be able to take this route out of poverty that includes the commercialization of small-scale farming.

### The Importance of Smallholders Becoming Business-Minded

Field veterinarians in northern Uganda and staff at the veterinary faculty commonly questioned smallholders' priorities and saw their small-scale livestock enterprises as a barrier to escaping poverty. Thus, as with the dominant discourse emerging from the policy documents, there was a strong idea among these informants that subsistence farming was problematic and that smallholders needed to be “sensitised” to think more in terms of business and entrepreneurship in order to succeed as farmers. Maxime from the veterinary faculty described this matter in terms of “*treating the psychology of farmers,”* implying that it is important to understand how farmers think for the purpose of changing their mindsets: “*As a vet in extension work, I mean, it's both about treating the psychology of farmers as well as treating the physical body of animals.”*

In this narrative, large-scale farming is both the main option and end goal for smallholder farmers. In the university, the veterinary curriculum had been adjusted to support this narrative in that there has been a greater focus in the last few years on business and entrepreneurship in the training of veterinary students. As a result, the training now concentrated more on the role of veterinarians in turning smallholders into market-driven and business-oriented entrepreneurs. It was believed that a shift in smallholders' mindsets would play a key role in achieving the envisioned transformation of the agriculture and livestock sector. In line with this narrative, several veterinary faculty respondents emphasized in interviews that it was important for veterinary students to have the ability to convey a business mindset to smallholders.

The same narrative was also found among field veterinarians, who commonly problematised smallholders' subsistence levels in rural areas, and emphasized the need for rural smallholders to become more like large-scale farmers closer to urban areas, who were believed to be better educated and both demand and market-driven. In sum, a discourse coalition of field veterinarians, faculty staff and policymakers could be identified that adhered to and supported a dominant discourse about upscaling, entrepreneurship and business orientation as a route out of poverty.

### The Simultaneous Challenge and Importance of Transferring Knowledge

The dominant discourse coalition described the transfer of knowledge as a central means for transforming smallholders' mindsets. The veterinary sector needs to develop ways of doing this that will enable farmers to become ‘sensitised' and farmers are required to contribute by taking part in the training opportunities offered to them and embracing the suggested approaches of entrepreneurship.

In interviews with field veterinarians and staff members at the veterinary faculty, it was evident that they faced challenges regarding the “transfer” of scientific knowledge to smallholders who often did not share their views on livestock production or relied on other sources of information. Field veterinarian Adrian illustrated this problem, saying that: “*To me, the biggest challenge is the farmers. Their way of thinking and their backward beliefs. I learnt things in school, but farmers hesitate to follow our advice; instead they want to use leaves and other stuff to treat their sick animals. It is this challenge that we* veterinarians *meet in the field, of communicating scientific facts to farmers who rely on their religion and traditions in the villages.”* In this discourse, science becomes the better way of knowing and, as a consequence, “religion and traditions” are constructed as a problem. While field veterinarian Adrian describes the challenge that he and his colleagues face in the mission to ‘change smallholders' mindsets', this quotation also reveals that their experience of such challenges does not lead veterinarians to question the wider discourse framing smallholders' mindsets as the key problem.

Both field veterinarians and veterinary faculty respondents repeatedly stated that if only smallholders could become “enlightened” and “sensitised,” they would become part of “modernity” and experience a transformation from subsistence to commercial agriculture. This narrative implied that the persistence of small-scale farming was a result of smallholders' unwillingness to adopt new information and technology.

In interviews with veterinary faculty staff, they commonly suggested that the challenge of changing smallholders' mindsets could potentially be reduced by boosting the practical skills of veterinary students during their studies. Faculty staff also noted that despite the curriculum expanding its practical elements in recent years, after graduation many students still experienced a gap between their theoretical studies and the practical characteristics of clinical work in rural areas. The boosting of the practical component was thus mainly seen as a means of getting smallholders to understand and adapt their practices in line with the “correct” information. This idea of knowledge as a fixed entity and of scientific knowledge as always better than other ways of knowing and acting was also found in how field veterinarians and veterinary faculty members discussed paraprofessionals' perceptions of animal disease, for example how they differentiated between their own scientific knowledge and “pseudo medicine,” “gambling,” and “lack of science” when discussing a perceived knowledge gap among paraprofessional actors. Here, the credibility of scientific knowledge was also an important way for veterinarians to legitimize their role in the veterinary sector.

### The Problem With “Quacks”

When staff at the veterinary faculty and field veterinarians identified challenges in the livestock sector, they often talked about individuals who called themselves veterinarians or paraveterinarians but had no formal qualifications, inadequate training or no knowledge of veterinary medicine at all. In interviews with veterinarians, they were referred to as quacks (i.e., a person performing quackery) and defined as someone performing veterinary work without the required competence or supervision by a professional. The actors described as quacks were individuals with limited or no training in animal health and medicine who provided incorrect advice or treatment under the pretense of being skilled in veterinary medicine, even calling themselves veterinarians. The veterinary informants believed that the liberalized market of veterinary pharmaceuticals, which means that almost all drugs can be bought over the counter without a prescription, had exaggerated the problem of quacks. Several field veterinarians and paraprofessionals in this study had witnessed the misuse of pharmaceuticals by actors in the field who worked by an approach of “trial and error” rather than relying on “evidence-based medicine.” The issue of quacks was perceived by the veterinary informants to be linked to underfunding of the veterinary sector and the unregulated training and supervision of paraprofessionals. Staff member Maxime at the veterinary faculty described this problem of paraprofessionals performing quackery, in particular when dealing with ASF in villages: “*Like with ASF, a major disease in the north, there are some local paravets who think it should be treated like any other common disease. In the end, they* [paraprofessionals, referred to by this informant as “paravets”] *themselves transmit it to several places. Farmers in the communities believe in them and that ASF should be treated because it is cheap and easy to access the treatment from paravets. Sometimes, it may look like the pigs recover, but it's a big challenge because the work of paravets instead makes things much worse.”* Staff members at the veterinary faculty and field veterinarians commonly perceived ASF to be one of the greatest threats to boosting pig production in the country, and furthermore a major constraint to the vision of transforming subsistence pig farming into commercial agriculture.

Overall, quacks were perceived to be a problem by all veterinary and paraprofessional informants, not only because they spread false information to smallholders and lead to animals being lost, but also because they undermine and contradict the work of field veterinarians and competent paraprofessionals. In this context it should be noted that while two paraprofessional informants were recommended for interviews based on smallholders and paraprofessionals classifying them as quacks, all the paraprofessionals in the study (including these two) distanced themselves from quacks.

Field veterinarian Amos explained the difficulty smallholders had in differentiating between animal health service providers and consequently how poor advice from a paraprofessional or quack would result in smallholders losing trust in the veterinary profession as a whole: “*Farmers can say “I called a vet,” but then an unqualified person comes to the village. So farmers assume that it was a vet that came. To a farmer, that person was a vet, but he wasn't really.”* Amos continued by describing how the faulty advice provided by untrained people (who smallholders perceived to be field veterinarians) caused problems for field veterinarians who then had to both solve emerging animal health issues as a result of the wrong advice and try to explain to farmers that the previous advice they had received was in fact incorrect: “*It's hard for us because we then need to do de-advice work.”*

### Local Perspectives Challenging the Dominant Discourse

#### Smallholders' Perspectives

As mentioned above, all the actors in this study agreed that the presence of field veterinarians in rural areas was limited. As can be seen in this section, this made smallholders turn to more affordable and locally available paraprofessionals.

Smallholders in this study generally combined crop and livestock production. Farming was often framed as a necessity to sustain their families, rather than something desirable or preferable in itself. Smallholder Morris, who engaged in crop and livestock production, dreamt about something other than farming when he envisioned his children's future: “*I don't want my children to follow in my footsteps. I want them to go to school, get a degree and then I hope they'll find good jobs. Digging in the garden is just for me, what I have to do, but my hope is that my children will be able to leave village life and farming, because if you leave the village you can get more opportunities in life. There are more possibilities in the cities compared to the village.”* In this context, livestock production was often inscribed with a hope of escaping farming. Upscaling livestock production was perceived as one of several strategies to increase the chances of a better life, eventually escape the countryside and live a modern life with a paid job in an urban area. Thus, according to several smallholders, upscaling livestock production was seen as a potential launch pad to a better life rather than an end goal in itself.

In contrast to cattle and goats that have longer histories in this area, pigs are not embedded in local traditions and are not used in witchcraft or dowries, which also means they can be sold more easily. Pig production was understood as a way of obtaining a quick return on investments as compared with, for example, poultry that have less economic worth, goats that produce fewer offspring with far longer in between, and cattle that are much more expensive and very rare in the villages after the most recent period of conflict. Pigs were repeatedly described by smallholders as “a shortcut to money.” Keeping pigs meant easy access both to cash, by selling off pigs, and to financial security as a buffer that could be used in times of need. Despite these benefits of pig production, many also complained about free-roaming pigs destroying crops, which added to already existing social tensions among neighbors. Another challenge was animal health issues. Many smallholders generally found it difficult to distinguish between different animal diseases, but the lack of experience of how to care for pigs made it even more difficult to interpret symptoms and signs of sickness in pigs. The uncertainty and frustration caused by animal disease was expressed well by smallholder Iris: “*I feel that there is not much to do when my animals get sick. How to know which drug to give them? They just die.”* When discussing disease in pigs, several smallholders recognized clinical signs that could be related to ASF, such as skin color changes, loss of appetite, fever and rapid death after showing the first signs of disease. However, few smallholders linked such clinical signs to ASF specifically, but rather referred to them as malaria, fever or “orere” (a Luo word for unspecified disease outbreaks). Smallholders believed that many livestock problems (including ASF) could potentially be solved by better access to veterinary care, but not everyone could spend money on such services due to the relatively high costs in relation to potential incomes from livestock production.

The smallholders in this study had very limited access to veterinary care and pharmaceuticals (Annex 4). The purchase of pharmaceuticals incurred the costs of traveling to a town or city, something that few were able to prioritize. Instead, most smallholders used locally available resources such as ash, leaves and washing powder to treat sick animals. Several smallholders believed that their pig production could be improved by constructing housing for their pigs (which is one important biosecurity measure to limit the spread of ASF as well as other diseases by limiting the intermingling of pigs from different households). Housing was seen as a good way to protect pigs from disease and also reduce social conflicts among neighbors due to the destruction of crops by free-roaming pigs. However, owing to more acute household needs and a lack of capital, few smallholders could prioritize such investments. Only 11 of the 101 survey respondents stated that they had the contact details of a veterinary actor and had contacted them for animal check-ups or consultations in the past 12 months. None of these veterinary actors was identified by the smallholders (or by the field assistant) as a professional veterinarian. Seven of these veterinary actors were identified as paraprofessionals with no or limited formal training in livestock production. The other four veterinary actors could not be identified either as a field veterinarian or a paraprofessional.

#### The Role and Perspectives of Paraprofessionals

The paraprofessionals in this study all worked and lived among smallholders in villages in Nwoya district. Thus, in contrast to field veterinarians who were based in towns and cities, paraprofessionals were closer and more easily accessible for smallholders than field veterinarians. All except one paraprofessional (who worked on livestock projects for a local NGO) worked privately and had limited contact with field veterinarians at sub-county level. In contrast to many of the field veterinarians active in the region, the paraprofessionals all belonged to the Acholi people and spoke Luo, which they recognized as being important for communicating with smallholders in the area.

The length and content of the training varied among paraprofessionals. Three were trained in animal production and management, with either a 2-year certificate or a 3-year diploma. Two had been trained in general agriculture, with a focus on crop production, but had joined the animal health sector due to work opportunities in rural areas. Lastly, one paraprofessional, Francis, had a BSc in human medicine. In contrast to the other paraprofessionals who classified themselves as “real vets,” Francis called himself a scientist. However, the fact that he had an education and kept more animals than most of the other villagers meant that he was often approached by nearby smallholders in need of help. While he was commonly perceived to be a veterinarian, for Francis, however, it was important to avoid being classified as a veterinarian or a paraprofessional as he did not want to be one of those individuals “*…doing bad things in the name of a veterinarian.”*

Francis can be seen as the exception that proves the rule: unlike him, paraprofessionals gained legitimacy and credibility by constructing themselves as veterinarians. This way of identifying themselves played a crucial role both in terms of how they perceived their own work and role and how they were perceived by smallholders. The paraprofessionals differentiated themselves from field veterinarians, referring to them as doctors. According to the paraprofessionals, the doctors spent a great deal of time reading books, but often lacked the knowledge important for veterinary work, such as knowledge of the culture and practices and understanding of the local conditions of smallholder farmers. Thus, the emphasis on understanding smallholders' contexts and having practical skills were important aspects for legitimizing their role in the veterinary sector. Nevertheless, it was also in relation to field veterinarians that paraprofessionals risked losing legitimacy and credibility. Two paraprofessionals in this study had experience of working with field veterinarians and recognized that their subjectivities shifted in this context. For example, one paraprofessional, George, said that he was not called “*a real veterinarian”* by his professional counterpart, but instead was referred to as “*my child.”* Paraprofessional Richard had a similar experience, explaining that he was called “*an assistant”* when he worked with a field veterinarian. It should be noted here that smallholders had no method for distinguishing between the various animal health service providers, and paraprofessionals were widely considered by smallholders to be veterinarians. However, if they failed to cure smallholders' animals, they were at risk of being classified as quacks—a group with which no one wanted to be associated.

All except one paraprofessional in this study had been trained in some aspects of entrepreneurship and business. However, in contrast to the dominant discourse, paraprofessionals did not make the connection that smallholders becoming more business minded would be a key route out of poverty. Rather, they saw this training as helpful in building their own businesses and become more entrepreneurial themselves. Paraprofessional Jacob described this focus of becoming more business-oriented in his own work: “*You have to be smart and plan your own business. It's important to know the market and try to modernize your work. You need to understand farmers and find ways to get good prices for treating animals in the communities.”* Even though paraprofessionals believed that it was important that they themselves became more business-minded, they did not imply that smallholders had to follow the same route. However, like field veterinarians and faculty staff, paraprofessionals also believed that it was important to transform the mindsets of smallholders, particularly in relation to how they understand animal disease. Here it was interesting to note that the majority of paraprofessionals treated clinical signs of ASF, and that three of them claimed to regularly vaccinate pigs as a prevention measure for this disease, even though there is currently no ASF vaccine on the market. In this context, changing the mindsets of smallholders was perceived to be important in order to make them more willing to pay for vaccines and pharmaceuticals supplied by paraprofessionals. Paraprofessional Jacob described this here: “*What I see from farmers is that they still have traditional mindsets. For instance, if pigs are suffering from ASF, they don't know how to use animal medicines to treat the particular disease that the medicine was created for. The tendency among farmers here is to use local techniques, to treat diseases like in the old days. Changing their mindsets is not easy. But they need to understand that ASF and other diseases need to be dealt with by using our medicines, but farmers always try to avoid the costs.”*

Even though the paraprofessionals questioned smallholders' mindsets in relation to how they dealt with animal disease, especially ASF, they did not assume that the scale of smallholders' livestock production was the main problem. Overall, paraprofessionals were more accepting of the current state of smallholders' small-scale enterprises compared with other veterinary actors, and they did not perceive it to be their role to foster them toward commercialization [cf (57), p. 3].

## Discussion

This study combined an investigation of the structure of the Ugandan veterinary sector and the availability of veterinary support to smallholders with a discourse analysis of how central actors in the sector framed the key challenges and solutions. The focus of the study was on northern Uganda, a region particularly dominated by past conflicts, with resulting high levels of poverty and marginalization (32), and on pigs, an animal that has become increasingly popular in the country and is promoted as a comparatively cheap and rapidly reproducing livestock, facilitating smallholder upscaling and poverty reduction (4, 28, 64, 65).

The findings revealed that many smallholders engaged in pig production with the hope of increasing their chance of escaping rural poverty. However, a lack of finance made investments difficult, and the burden of diseases diminished the financial potential of pig production. Furthermore, the findings showed that all the informants, who are formally and informally involved in the Ugandan veterinary sector from national to local level, agreed that underfunding in the sector created major problems for service delivery. This is a general tendency in agricultural development in Africa, where downsizing of the public sector and privatization of extension services have led service providers to turn to the wealthier farmers who can pay, leaving smallholders behind (66–68).

There was less consensus in how the various actors constructed the causes and solutions to the overarching problem of a lack of veterinary support for smallholders. A dominant discourse, supported by a discourse coalition of policymakers, veterinary faculty staff and field veterinarians, constructed upscaling and commercialization of agriculture as the route out of poverty for smallholders. They believed that this would necessitate a change in smallholders' mindsets to make them more business-minded and entrepreneurial. In this context, smallholders were positioned as “backwards” and in need of being “sensitised” in order to be willing to conform to a narrative where upscaling and modernization of livestock production are seen as the key to escaping poverty. A plethora of studies on agricultural development in Africa in recent years reflects a similar picture of dominant discourses of agricultural development in effect turning a blind eye to structural reasons behind the downscaling of the public sector, instead framing smallholders' lack of entrepreneurial will as the main problem (37, 69–73). Such constructions of smallholders being the cause of, and thus also bearing responsibility for, their own poverty are ahistorical and apolitical, and serve to uphold and support dominant neoliberal narratives (14, 32, 69, 74). Previous research further demonstrates that intervention strategies focusing mainly on attitudinal and behavioral change have a limited impact, particularly in poverty-ridden contexts, if underlying structural inequalities are not properly addressed (75–78). The focus on individual attitudes also diverts attention away from these structural inequalities. In contrast to the dominant discourse of the upscaling of farming as the ultimate goal for poor smallholders, our findings showed how smallholders' narratives of development and the role of livestock production are diverse, but often frame upscaling livestock production as a launch pad to a better life outside farming rather than an end goal in itself. In this context, it is important to note that numerous studies show that many young people in Africa aspire to a future outside the agricultural sector and have other hopes than becoming farmers (79–83). However, despite evidence of this also being the case among smallholders in the present study, there was no acknowledgment of this in the dominant discourse [cf (84)].

This study, like previous ones (9, 11, 44) showed that paraprofessionals fill an important gap in veterinary service provision as there are too few qualified veterinarians. Those that are qualified are often reluctant to work in rural areas due to the small profits involved (9). The services of paraprofessionals are usually more affordable and more accessible to smallholders than those of qualified veterinarians (4, 9, 28). At the same time, our findings highlighted important knowledge gaps in livestock management and animal disease among some paraprofessionals, occasionally resulting in severe negative effects for both animal welfare and smallholder livelihoods. Incorrect advice and treatment could, for example, contribute to the spread of ASF. Closer collaboration between veterinarians and paraprofessionals could be an important part of a solution to this, starting with recognition by veterinarians and paraprofessionals of the complementarity and value of each other's competence. Several studies have shown the important role that paraprofessionals can play in disease prevention and even eradication (85–87). A second component for paraprofessionals to play a key role in disease prevention and eradication is revived and standardized training of paraprofessionals, something that is also emphasized in other studies (88, 89). Smallholders also need to have strategies to distinguish between the relative qualifications of the various veterinary actors providing services (90).

A weakness of the present study is the limited number of veterinary and paraprofessional informants involved and the fact that, due to travel restrictions during the Covid-19 pandemic, it was not possible to conduct interviews with faculty staff and paraprofessionals (except one) in person. This was mitigated by drawing more heavily on policy documents in the discourse analysis, discussing findings with key informants, and ensuring that the conclusions did not go beyond what the data supported.

## Conclusions

A lack of qualified veterinarians in Uganda and smallholders' inability to pay for them has led to paraprofessionals with varying levels of training being key providers of animal healthcare advice to smallholders in rural northern Uganda. This currently entails risks to animal health. The findings of this study revealed that veterinary work and policy are dominated by a discourse that emphasizes smallholders' lack of an entrepreneurial mindset and paraprofessionals' lack of knowledge in livestock management and disease as key reasons for smallholders failing to upscale their livestock production and escape poverty. The discourse ignores the underlying structural reasons for this situation and overlooks paraprofessionals' competence and the possibility that they could play an important role in the provision of animal health care. Paraprofessionals are often familiar with smallholders and their environments, and thus have extensive knowledge of the local conditions of livestock production in rural areas. As such, they have important practical skills and knowledge when it comes to smallholders' needs and an awareness of the options available to them.

In conclusion we have identified three factors as key to improving access to good quality veterinary support for smallholders in this context:

1. revived certified training of paraprofessionals
2. strategies to help smallholders interpret the different competences of actors working in livestock management and health so that they can identify and reject poor advice
3. improved collaboration and communication between veterinarians and paraprofessionals.

## Data Availability Statement

The raw data supporting the conclusions of this article will be made available by the authors, without undue reservation.

## Ethics Statement

The studies involving human participants were reviewed and approved by Makerere University College of Health Sciences (ref 2019-062). Written informed consent for participation was not required for this study in accordance with the national legislation and the institutional requirements.

## Author Contributions

AA, KF, KH, EC, and SS-L: study design and methodology. AA: manuscript original draft and data collection with support from KF, KH, EC, and SS-L. AA, KF, and KH: conceptualization. KL, KH, EC, and SS-L: reviewing and editing of all versions of the draft. All authors have read and approved the final manuscript.

## Funding

This research was funded by the Swedish Research Council VR Grant No. 2017-05518.

## Conflict of Interest

The authors declare that the research was conducted in the absence of any commercial or financial relationships that could be construed as a potential conflict of interest.

## Publisher's Note

All claims expressed in this article are solely those of the authors and do not necessarily represent those of their affiliated organizations, or those of the publisher, the editors and the reviewers. Any product that may be evaluated in this article, or claim that may be made by its manufacturer, is not guaranteed or endorsed by the publisher.

## Footnotes

^1^The fact that pigs are appreciated for these particular material qualities is also interesting in itself, and points to how different animals with their diverse materialities and relations with humans have the ability to have different impacts on human societies, but is not explored further in this article.
