# Supplementary material for: Diverging Discourses: Animal Health Challenges and Veterinary Care in Northern Uganda
Source: Front Vet Sci. 2022 Mar 10;9:773903. doi: 10.3389/fvets.2022.773903 (PMC8960384; doi:10.3389/fvets.2022.773903)
Supplement: Supplementary file 1 [file Data_Sheet_1.zip › Annex 1.DOCX]

# Annex 1

# Interview guide: Faculty staff at Makerere University

Interviews via Zoom 2020/2021

## Background information

Name:

Age:

Home district:

Mother tongue:

Educational background:

Current position:

## Working with veterinary education

1. What do you enjoy most about your work?
2. What are some of the challenges you face in your work?
3. How would you describe a “typical” veterinary student at Makerere? (For example: gender, age, home district, occupational background of parents.)
4. What subjects and courses are most popular among students in the veterinary school?
5. What kinds of jobs do you think most veterinary students want to apply for after graduation?
6. To your knowledge, how has the veterinary programme at Makerere University changed and developed over time?

## Veterinary profession (with focus on farm animals)

1. Other than knowing about animal diseases, what other things do you think are important for veterinarians to know about?
2. Can you please describe how the work of District Veterinary Officers (DVOs) and veterinary officers is structured in Uganda?
3. In general, how much training in extension work do veterinary students get at Makerere?
4. What are some challenges veterinarians can face when working with farmers?
5. When I interviewed veterinarians in Northern Uganda, they often mentioned challenges with “fake vets” (sometimes also described as “quack vets”). Have you also heard about so-called “fake vets”? If so, can you please describe how they operate?
