# Supplementary material for: Diverging Discourses: Animal Health Challenges and Veterinary Care in Northern Uganda
Source: Front Vet Sci. 2022 Mar 10;9:773903. doi: 10.3389/fvets.2022.773903 (PMC8960384; doi:10.3389/fvets.2022.773903)
Supplement: Supplementary file 1 [file Data_Sheet_1.zip › Annex 2.DOCX]

**Annex 2.** Results from Participatory Rural Appraisal ranking exercises conducted in village A in 2019. The village is located in Nwoya district, northern Uganda. During the rankings, the participants described problems related to specific animal species, suggested ways to prevent or solve these problems, and then ranked the problems in relation to each other. The scores presented in “total” represent the sum of scores for all groups that participated and for each animal species included in the ranking. The higher the score, the larger the problem, as perceived by the participants, and vice versa.

|  | PRA 1 | PRA 2 | PRA 3 | PRA 4 | PRA 5 | Total |
| --- | --- | --- | --- | --- | --- | --- |
| No. participants |  | | | | |  |
| Total | 9 | 9 | 6 | 10 | 9 | 43 |
| Male | 0 | 0 | 3 | 5 | 7 | 15 |
| Female | 9 | 9 | 3 | 5 | 2 | 28 |
|  |  |  |  |  |  |  |
| Average age | 33 | 35 | 36 | 33 | 30 | 33 |
| Animals owned/managed by participants  Rank chickens | Chickens, ducks, goats, pigs | Chickens, cattle, goats | Chickens, cattle, goats, pigs | Chickens, goats, (pigs in the past) | Chickens, goats |  |
| Disease outbreak  Cough  Access to vets*  Insect around eyes  Wild animals  Theft  Jealousy  Tiny insects  Liver disease  Run around  Lack of feed  Road accident | 9  8  3  7  5  4  2  6  N/A  N/A  1  N/A | 9  7  8  5  3  2  1  N/A  6  4  N/A  N/A | N/A^2^  N/A  N/A  N/A  N/A  N/A  N/A  N/A  N/A  N/A  N/A  N/A | 7  6  2  5  N/A  3  4  N/A  N/A  N/A  1  N/A | 9  5  6  N/A  8  7  4  3  N/A  N/A  N/A  1 | 34  26  19  17  16  16  11  9  6  4  2  1 |
|  |  |  |  |  |  |  |
| Other problems |  | Swollen head, worms in stomach |  |  |  |  |
| Rank goats |  |  |  |  |  |  |
| Access to vets*  Diarrhoea  Jealousy | 6  8  2 | 8  4  5 | N/A  N/A  N/A | 5  7  2 | 8  5  7 | 27  24  16 |
| Worms | 7 | N/A | N/A | 6 | 1 | 14 |
| Skin disease  Cry and die  Theft  Eat plastic mat  Flies  Cough  Overeat feed  Crops  Saliva mouth  Other problems  Rank pigs  Disease outbreak  Jealousy  Access to vets*  Crops  Access water  Cough  Ticks  Jiggers  Road accident  Other problems | 5  4  N/A  N/A  3  N/A  1  N/A  N/A  Men spend livestock money on alcohol  N/A  N/A  N/A  N/A  N/A  N/A  N/A  N/A  N/A | 3  7  6  N/A  N/A  N/A  N/A  2  1  Swollen neck, body becomes thin until they die. Men spend livestock money on alcohol  N/A  N/A  N/A  N/A  N/A  N/A  N/A  N/A  N/A | N/A  N/A  N/A  N/A  N/A  N/A  N/A  N/A  N/A  4  6  7  5  N/A  3  2  1  N/A | 4  3  N/A  N/A  N/A  N/A  1  N/A  N/A  Better access to vets would solve animal health issues  N/A  N/A  N/A  N/A  N/A  N/A  N/A  N/A  N/A | 2  N/A  6  4  N/A  3  N/A  N/A  N/A  Foot and mouth disease  7  5  3  4  6  2  N/A  N/A  1  Theft | 14  14  12  4  3  3  2  2  1  11  11  10  9  6  5  2  1  1 |

*Access to vets also includes access to pharmaceuticals.

^2^ N/A = Not available. Each group decided what animal species to include in the ranking exercise based on their own preferences. Except for one group that included all animal species in the ranking, the groups choose to rank problems related to either one or two animal species. Since groups identified and described problems related to specific animal species independent of each other, problems included in the ranking vary between the groups to some degree.
