# Supplementary material for: Diverging Discourses: Animal Health Challenges and Veterinary Care in Northern Uganda
Source: Front Vet Sci. 2022 Mar 10;9:773903. doi: 10.3389/fvets.2022.773903 (PMC8960384; doi:10.3389/fvets.2022.773903)
Supplement: Supplementary file 1 [file Data_Sheet_1.zip › Annex 3.DOCX]

# Annex 3

## Survey: smallholders

Northern Uganda 2019

Village name: ............................. Household No: .............. Date of interview: ..................

Interviewed by (name): .................. Name of person(s) interviewed: ...................................

*Ask if there is anyone at home who is involved in farming. If not, go back later.*

### BACKGROUND INFORMATION

1) Who are you speaking to? (circle more than one if appropriate):

Man (age): ..... Woman (age)...........

2) Who lives in this household? (only those who live in it, not those belonging to family but living somewhere else)

Number of adults (over 18): .......... Number of children (under 18): .............

3) Who does the farming in this household? (circle more than one if appropriate)

Man Woman Children

### LIVESTOCK

Which animal(s) do you have? (circle)

For chickens, goats and pigs: rank the problems (*biggest problem is 1 and smallest problem is 5):*

GWENO (chicken): YES NO

.......... (1, 2, 3, 4 or 5) NYEKO/KWOR (jealousy/thieves)
.......... (1, 2, 3, 4 or 5) ORERE (outbreaks)

.......... (1, 2, 3, 4 or 5) TOK (small insects around the eyes)

.......... (1, 2, 3, 4 or 5) AONA (coughing)
.......... (1, 2, 3, 4 or 5) DAKTA PE (no veterinarian to ask for help)

*.......... (1, 2, 3, 4, 5 or 6) MUKENE (other, specify): .............................*

MUKENE (other): .......................................................................................................................

DYEL (goat): YES NO

.......... (1, 2, 3, 4 or 5) NYEKO/KWOR (jealousy/thieves)

.......... (1, 2, 3, 4 or 5) CADO PIKWIDI (diarrhoea caused by worm)

.......... (1, 2, 3, 4 or 5) AONA (coughing)
.......... (1, 2, 3, 4 or 5) OCELCEL (crying and then die)
.......... (1, 2, 3, 4 or 5) DAKTA PE (no veterinarian to ask for help)

*.......... (1, 2, 3, 4, 5 or 6) MUKENE (other, specify): .............................*

MUKENE (other): ......................................................................................................................

OPEGO (pig): YES NO

.......... (1, 2, 3, 4 or 5) NYEKO/KWOR (jealousy/thieves)
.......... (1, 2, 3, 4 or 5) ORERE (outbreaks)
.......... (1, 2, 3, 4 or 5) DAKTA PE (no veterinarian to ask for help)

.......... (1, 2, 3, 4 or 5) PEKECAM (lack of feed)

.......... (1, 2, 3, 4 or 5) BALOJAMI (destroying crops)

*.......... (1, 2, 3, 4, 5 or 6) MUKENE (other, specify): .............................*

MUKENE (other): ........................................................................................................

DYANG (cattle): YES NO
ROMO (sheep): YES NO
ATUDO (duck): YES NO
Others (specify which animal): ....................................................................................................

### VETERINARIANS

4) a) Do you have a phone number for a veterinarian? Yes No

b) **If YES**: Do you know the name of the veterinarian? ..................................................

c) Have you been in contact with a veterinarian this year? Yes No

d) **If YES:** How many times have you contacted a veterinarian this year? ................... times

e) **If YES**: Why did you contact a veterinarian (what was the problem)? ......................................................................................................... ........................................................................................................

f) **If YES:** What was your experience of your contact with the veterinarian? ........................................................................................................ ........................................................................................................

g) **If YES:** How much money did you pay the veterinarian? ................................... UGX

h) How much money have you spent on drugs this year (if you bought them from a drug shop and not from the veterinarian)?

................................... UGX
