# Supplementary material for: Diverging Discourses: Animal Health Challenges and Veterinary Care in Northern Uganda
Source: Front Vet Sci. 2022 Mar 10;9:773903. doi: 10.3389/fvets.2022.773903 (PMC8960384; doi:10.3389/fvets.2022.773903)
Supplement: Supplementary file 1 [file Data_Sheet_1.zip › Annex 4.DOCX]

**Annex 4. Survey conducted in study villages A and B (Nwoya district, northern Uganda) at the end of December 2019.**

**A.** Demographic data of respondents in a survey of smallholders’ contact with veterinarians/paraprofessionals, animal species and ranking exercise conducted in study villages A and B at the end of December 2019. HH refers to household

|  | Men* | Women* | Total* |
| --- | --- | --- | --- |
| Respondents |  |  |  |
| (No.) | 64 | 31 | 101 |
|  |  |  |  |
| Age (years)  Average (min-max) | 41 (19–69) | 40 (22–58) | 41 (19–69) |
| Adults in HH (no.)  Average (min-max)  Children in HH (no.)  Average (min-max) | 3.1 (1–8)  3.7 (0–13) | 3.2 (1–8)  4.1 (1–8) | 3.2 (1–8)  3.9 (0–13) |

* Six respondents did not state their gender or age

**B.** Results relating to veterinary services in a survey of smallholders’ contact with veterinarians/paraprofessionals, animal species and ranking exercise conducted in study villages A and B at the end of December 2019. PP refers to paraprofessional and vet refers to veterinarian.

| Questions | Men | Women | Unspecified gender | Comments | Total | |
| --- | --- | --- | --- | --- | --- | --- |
| Have a vet/pp actor’s telephone number (no.) | 5 | 4 | 2 |  | 11 |  |
| Type of actor^1^ |  |  |  | PP 1* (3),  PP 2 (2),  Unknown 1 (2),  PP 3 (1),  PP 4 (1),  Unknown 2 (1), Unknown 3 (1) |  |  |
| Have contacted a vet/pp actor in the last 12 months (no.) | 5 | 4 | 2 |  | 11 |  |
| Have contacted a vet/pp actor in the last 12 months (no. of times)  Average (min-max) | 22 (4-48) | 5 (1-12) | 7 (2-12) |  | 13 (1-48) |  |
| Reasons for contacting a vet/pp |  |  |  | Check/treat animals. Respondents specifically mentioned problems due to tsetse flies, ticks, cough and diarrhoea as reasons for contacting vet |  |  |
| Experience of contact with a vet/pp^2^  Positive  Negative  Neutral | 2  2  1 | 4  0  0 | 2  0  0 | improved animal health  high costs, animal health not improved | 8  2  1 |  |
| Total costs of vet/pp services in the past 12 months (USD)  Average (min-max)  Purchase of animal medicines in the past 12 months (no.)  Total cost of animal medicines in the past 12 months (USD)  Average (min-max) | 199.9  33.3 (2.8-168.4)  10  613.4  61.3  (2.8-280.7) | 50.5  25.2  (5.6-44.9)  4  126.3  31.5 (11.2-47.7) | 11.2  5.6 (2.8-8.4)  2  233.0  116.5 (8.4-224.6) |  | 260.7  21.3 (2.8-168.4)  16  972.7  69.7 (2.8-280.7) |  |
|  |  |  |  |  |  |  |

* This specific paraprofessional actor was one of the field assistants who had no formal training in animal health or livestock production

^1^ The number in brackets indicates the number of respondents mentioning each actor

^2^ Experience here refers to answers by the 11 individuals that stated that they had contacted a veterinary/paraprofessional actor in the past 12 months

**C.** Animal species owned/managed by respondents in a survey of smallholders’ contact with veterinarians, animal species and ranking exercise conducted in study villages A and B at the end of December 2019

| **Animal species** | **Men* (no. of respondents keeping species)** | **Women* (no. of respondents keeping species)** | **Total (no. of respondents keeping species)** |
| --- | --- | --- | --- |
| Poultry | 56 | 27 | **88** |
| Goats | 43 | 14 | **59** |
| Pigs | 16 | 6 | **23** |
| Ducks | 13 | 6 | **20** |
| Cattle | 9 | 3 | **13** |
| Sheep | 10 | 2 | **12** |
| Other | 2 | 2 | **4** |

*Six respondents did not state their gender

**D.** Ranking. Smallholders in study villages A and B were asked to rank five suggested problems with animal species that they had in their household: goats, pigs and poultry. Those who wanted to were given the option to add other problems and include them in the ranking. After discussing all the problems for the respective animal species, respondents ranked the problems in relation to each other (1-5), with 5 indicating the largest problem and 1 indicating the smallest problem

| **Animal** | **Problem** | **No. respondents^1^** | **Women*** | **Men *** | **Total ranking score^2^** | **Average men** | **Average women** | **Average total** |
| --- | --- | --- | --- | --- | --- | --- | --- | --- |
| Chicken | Disease outbreak | 88 | 27 | 55 | 400 | 4.5 | 4.6 | 4.5 |
| Chicken | Cough | 88 | 26 | 56 | 298 | 3.5 | 3.0 | 3.4 |
| Chicken | Jealousy and theft | 75 | 23 | 49 | 257 | 3.0 | 4.0 | 3.4 |
| Chicken | Access to vet | 82 | 26 | 52 | 220 | 2.8 | 2.4 | 2.7 |
| Chicken | Insect eye | 50 | 13 | 37 | 76 | 1.4 | 2.0 | 1.5 |
| Goat | Diarrhoea | 57 | 14 | 41 | 256 | 4.5 | 4.4 | 4.5 |
| Goat | Cough | 55 | 13 | 40 | 196 | 3.6 | 3.5 | 3.6 |
| Goat | Access to vet | 54 | 14 | 39 | 165 | 3.0 | 3.2 | 3.1 |
| Goat | Jealousy and theft | 37 | 11 | 26 | 112 | 3.1 | 2.9 | 3.0 |
| Goat | Cry and die | 34 | 6 | 28 | 64 | 1.9 | 2.0 | 1.9 |
| Pig | Jealousy | 23 | 6 | 16 | 92 | 3.8 | 4.5 | 4.0 |
| Pig | Access to vet | 23 | 6 | 16 | 82 | 3.6 | 3.3 | 3.6 |
| Pig | Disease outbreak | 23 | 6 | 16 | 81 | 3.4 | 3.7 | 3.5 |
| Pig | Destroy crops | 23 | 6 | 16 | 70 | 3.2 | 2.7 | 3.0 |
| Pig | Lack of feed | 23 | 6 | 16 | 23 | 1.0 | 1.0 | 1.0 |

**Some respondents did not state their gender: six respondents ranking problems with chicken, two respondents ranking problems with goats and one respondent ranking problems with pigs did not state their gender

^1^Only respondents who had particular animal species in their household ranked problems relating to that species; not all respondents ranked all five suggested problems

^2^Sum of all ranking scores for each problem from all respondents who had ranked problems for that animal species
